# Morphometric Differences of Vocal Tract Articulators in Different Loudness Conditions in Singing

**DOI:** 10.1371/journal.pone.0153792

**Published:** 2016-04-20

**Authors:** Matthias Echternach, Fabian Burk, Michael Burdumy, Louisa Traser, Bernhard Richter

**Affiliations:** 1 Institute of Musicians’ Medicine, Freiburg University Medical Center, Breisacher Str. 60, 79106 Freiburg, Germany; 2 Department of Medical Physics, Radiology, Freiburg University Medical Center, Breisacher Str. 60, 79106 Freiburg, Germany; 3 Department of Otorhinolaryngology, Freiburg University Medical Center, Kilianstr. 5, 79106 Freiburg, Germany; Utrecht University, NETHERLANDS

## Abstract

**Introduction:**

Dynamic MRI analysis of phonation has gathered interest in voice and speech physiology. However, there are limited data addressing the extent to which articulation is dependent on loudness.

**Material and Methods:**

12 professional singer subjects of different voice classifications were analysed concerning the vocal tract profiles recorded with dynamic real-time MRI with 25fps in different pitch and loudness conditions. The subjects were asked to sing ascending scales on the vowel /a/ in three loudness conditions (comfortable = mf, very soft = pp, very loud = ff, respectively). Furthermore, fundamental frequency and sound pressure level were analysed from the simultaneously recorded optical audio signal after noise cancellation.

**Results:**

The data show articulatory differences with respect to changes of both pitch and loudness. Here, lip opening and pharynx width were increased. While the vertical larynx position was rising with pitch it was lower for greater loudness. Especially, the lip opening and pharynx width were more strongly correlated with the sound pressure level than with pitch.

**Conclusion:**

For the vowel /a/ loudness has an effect on articulation during singing which should be considered when articulatory vocal tract data are interpreted.

## I. Introduction

In recent years, interest in the analysis of the phonation apparatus using real time dynamic MRI technology has increased in both voice and speech research [1–6]. With regard to singing voice physiology, it has been shown that the vertical larynx position can change during phonation [7]. Furthermore, vocal tract shape has been evaluated. Here, it was shown that the vocal tract might be modified with respect to vocal registers [8–11] or might be adjusted in order to match vocal tract resonances or formants with voice source partials [12,13]. According to Titze et al. the term vocal tract resonances is used if the transfer function of the vocal tract is characterized whereas the term formant is used when the vocal tract is excited by a voice source [14]. These studies included the modification of different variables, such as pitch (perceptual term) and fundamental frequency (ƒ_o_, acoustical parameter) [9,12,13,15], vowel conditions [10,13,16], register [8–10,17] or different singing styles [18–20]. Different loudness (perceptual term) or sound pressure level (SPL, acoustical parameter) conditions have been neglected in most studies so far: the loud noise which is produced by the scanner prevented detailed analyses of the SPL. However, it could be hypothesized that SPL might influence articulation independent of register, ƒ_o_ or vowel condition.

In principle, there are three general strategies of SPL control in the voice. First, the subglottic pressure could be considered as the most important factor [21–24]. Here, increased subglottic pressure is associated with an increased SPL [23]. Secondly, the phonation type (such as breathy, flow phonation, normal, and pressed) and the associated grade of adduction of the vocal folds might contribute to the SPL [24–26]: flow phonation shows the greatest maximum flow declination rate which is associated with the greatest SPL [27]. Finally, resonatory properties of the vocal tract might affect sound pressure level, usually promoting the voice source partial which will be the strongest partial of the radiated spectrum and therefore determining SPL [27]. This is of importance especially for the lower resonances. It has been shown that the first vocal tract resonance is raised by a greater lip and jaw opening which could influence SPL [27]. Furthermore, the tracking of voice source partials by vocal tract resonances (often denoted as formant or vocal tract resonance tuning [28–30]) has been found to be of importance, especially for soprano voices [28,30,31] since this technique is considered to increase SPL. This tuning is more relevant for female singers`voices but not for speech [32]. The employment of resonance tuning by male professional singers has recently been discussed divergently in literature [29,33,34]. Furthermore, especially in professional singers, modifications of the lower vocal tract might produce a clustering of the vocal tract resonances 3 to 5 [35,36]. As a result, the spectrum partials in this region are boosted. In the case of individual singers such partials could be strongest in the voice spectrum with the consequence that the SPL is determined by these partials. These three control mechanisms are not thought to be independent [37]. However, in a recent study by Herbst et al. it was shown for the singing voice that vocal loudness could be independently controlled from glottal configuration [21].

As stated above the vocal tract could have a direct influence on SPL. However, indirect influences of the vocal tract on the SPL and loudness could also be possible: if there is a rise of subglottic pressure, changes of muscular activity concerning tension and/or adduction are expected at the glottal level [38,39]. The degree of glottal adduction is associated with the vertical larynx position, i.e. that a small abduction is found for a lower vertical laryngeal position [27]. For trained singers both x-ray studies [40] and photographical recordings [41] showed a lower vertical larynx position for greater loudness and effort conditions. A lowering of the vertical laryngeal position would have the effect of vocal tract elongation and therefore a decrease in vocal tract resonance frequencies, which is frequently denoted as *covered voice*, *voix sombrée*, *gedecktes Singen*. Such a lowering could be caused by an activation of external laryngeal strap muscles. The activation of these muscles, such as the sternothyroid muscle, could on the one hand lower the vertical laryngeal position and on the other hand facilitate a shortening and thickening of the vocal folds [42] which might influence vocal fold oscillatory patterns and therefore loudness.

However, changes of vocal tract shape and associated resonances could also have another effect on the voice source. From non-linear dynamic theory, it could be expected that the vocal tract interacts with the flow pulse [43,44] and/or the vocal fold oscillations [44,45]. Since the SPL is related to the transglottal airflow depending on transglottal pressure difference [27,32], a reverse effect of the vocal tract configuration on sound pressure level could be expected.

Due to the increased noise exposure there is very little articulatory data of the vocal tract achieved by MRI which reflects different loudness or sound pressure conditions. In their early MRI studies, Neuschäfer-Rube et al. [46] were not able to observe great differences concerning the vertical laryngeal position in relation to different loudness conditions. However, the same research group found changes in the oro-pharyngeal part of the vocal tract [47]. Since the MRI noise was not cancelled in these early studies and the perceptual term loudness was not objectified by means of SPL evaluations, the accuracy of the task performed by the subjects could not be verified. Furthermore, at the time of their studies, no dynamic real-time MRI recordings were possible.

As a conclusion, the role of the vocal tract in relation to loudness and SPL remains unclear. This study aims to analyse vocal tract shape differences associated with different loudness conditions in professional singers concerning their singing voice. It is hypothesized that there are articulatory differences for various loudness conditions, which would imply that professional singers modify vocal tract resonances to achieve loudness.

## II. Material and Methods

After approval from the local ethics committee (Freiburg University Hospital Ethical committee, Nr. 206/09, all subjects gave their written informed consent) 12 subjects were included in this study. All subjects are professional western classically trained operatic singers. None of the subjects had voice complaints and vocal fold pathologies were excluded by means of videostroboscopy and/or high speed digital imaging. A list of the subjects with their respective voice classification and taxonomy is provided in Table 1.

**Table 1:** Subjects, voice classification and taxonomy according to Bunch and Chapman [61].

Subject	classification	taxonomy
1	Soprano	2.1/2.4
2	Soprano	2.1/2.4
3	Soprano	3.15b1/3.17
4	Mezzo	2.1/2.4
5	Mezzo	2.1/2.4
6	Mezzo	2,4
7	Tenor	2.4/2.1
8	Tenor	2.1/2.4
9	Tenor	2.4/3.1a
10	Baritone	1.1/2.4
11	Baritone	1.1/2.4
12	Baritone	2.4/3.1a

The subjects were asked to sing an ascending diatonic major scale on the vowel /a/ (baritones: G3-E4 (196-330Hz), tenors: C4-A4 (262-440Hz), mezzo-sopranos: G3-E4 (196-330Hz), sopranos: A4-A5 (440-880Hz)). The rather low pitch for the mezzo soprano voice was chosen in order to make articulatory data comparable to male professional altos, analysed in a previous investigation [17]. Each pitch was to be sustained for approximately one second. This scale was repeated in three different loudness conditions and always in the same order: (1) in comfortable loudness (mf), (2) soft loudness (pp), and (3) great loudness (ff), respectively. Although there might be an effect on the results due to this order, it was firstly chosen to demonstrate both, pp and ff data relative to the comfortable loudness condition and secondly to exclude the effect that soft phonation might be altered by a great vocal loading after the ff task.

Comparable to our previous studies [8–10,15,19] the images display the mid-sagittal plane. The recordings were performed using the 3.0 T TIM TRIO (Siemens, Germany) MRI device with the subject in the supine position with 25 frames per second [11,48]. Therefore, each pitch of the diatonic scale was represented by approximately 25 frames. The exact MRI parameters used are provided in the study by Burdumy et al. [48].

Also in analogy with our previous experiments the audio signal was recorded with an optical microphone system (CONFON HP-SI 01, MR confon GmbH, Magdeburg, Germany), that included two microphone recordings (one recording vocalizations and background MRI scanner noise and one recording scanner noise only). During the recording the subjects were provided with the audio signal over headphones as acoustic feedback. Two experts (both otolaryngologists and singers) checked if the subjects performed the desired task. Only sequences found acceptable by the experts as well as by the singers themselves were subsequently analysed.

**Fig 1:**
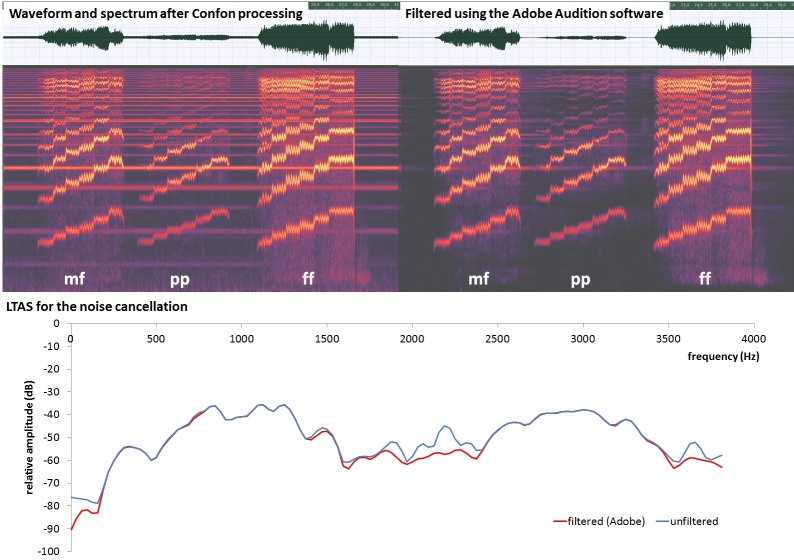
Waveform (upper row) and audio spectrum (middle row) for the file after the first noise cancellation (Confon, left panel) and after the second noise cancellation (Adobe, right panel). The waveforms include all three loudness tasks (mf = mezzoforte, pp = pianissimo, ff = fortissimo, respectively). The lowest row shows Long Time Average Spectra (LTAS) for both files, after the first noise cancellation (blue) and the second noise cancellation (red), respectively.

In order to verify that the intended perceptual loudness condition was fulfilled by the subjects, the audio recordings of each task were analyzed with respect to the SPL after noise cancellation. The first step in cancelling the scanner noise was performed automatically using dedicated software (Digital Audio Presentation Center, CONFON DAP-center mkII+, MR confon GmbH, Magdeburg, Germany). After this first noise cancellation the signal of the scanner noise was still present in most audio signals. In order to cancel this scanner noise, a part of the audio recording was analyzed where only the scanner noise but not the voice was present. This part was marked as reference noise and cancelled throughout the entire audio file using the Adobe Audition Software (Adobe Systems, San Jose, CA, USA). All tasks (pp, mf, ff, respectively) were filtered using the exact same filter properties. Fig 1 shows long time average spectra and spectra for a single task recording after the first cancellation by the Confon system and after the second noise cancellation procedure using the Adobe software. Both related audio files (S1 Audio and S2 Audio) are also provided as supplementary material. The SPL measurement was performed using the Praat software (University of Amsterdam).

**Fig 2:**
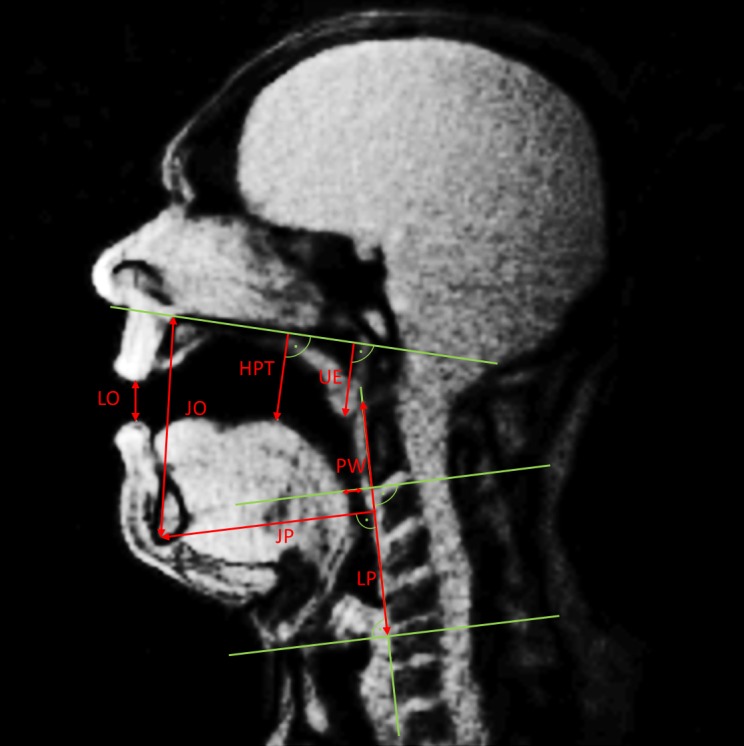
Measured distances in each frame of the MRI material (red arrows), as described previously in detail [8–11,15]. The auxiliary lines are shown in green. LO (Lip Opening), JO (Jaw Opening), JP (Jaw Protrution) HPT (Highest Point Tongue), UE (Uvula Elevation), LP (vertical Larynx Position).

In each MR frame of the MRI material a series of measures were taken (Fig 2), as described in previous studies [8–11,15]: lip and jaw opening, height of the tongue, jaw protrusion, pharynx width, uvula and larynx position. Mean values were calculated for each pitch (mean values across approximately 25 single values per pitch) and related to ƒ_o_.

**Fig 3:**
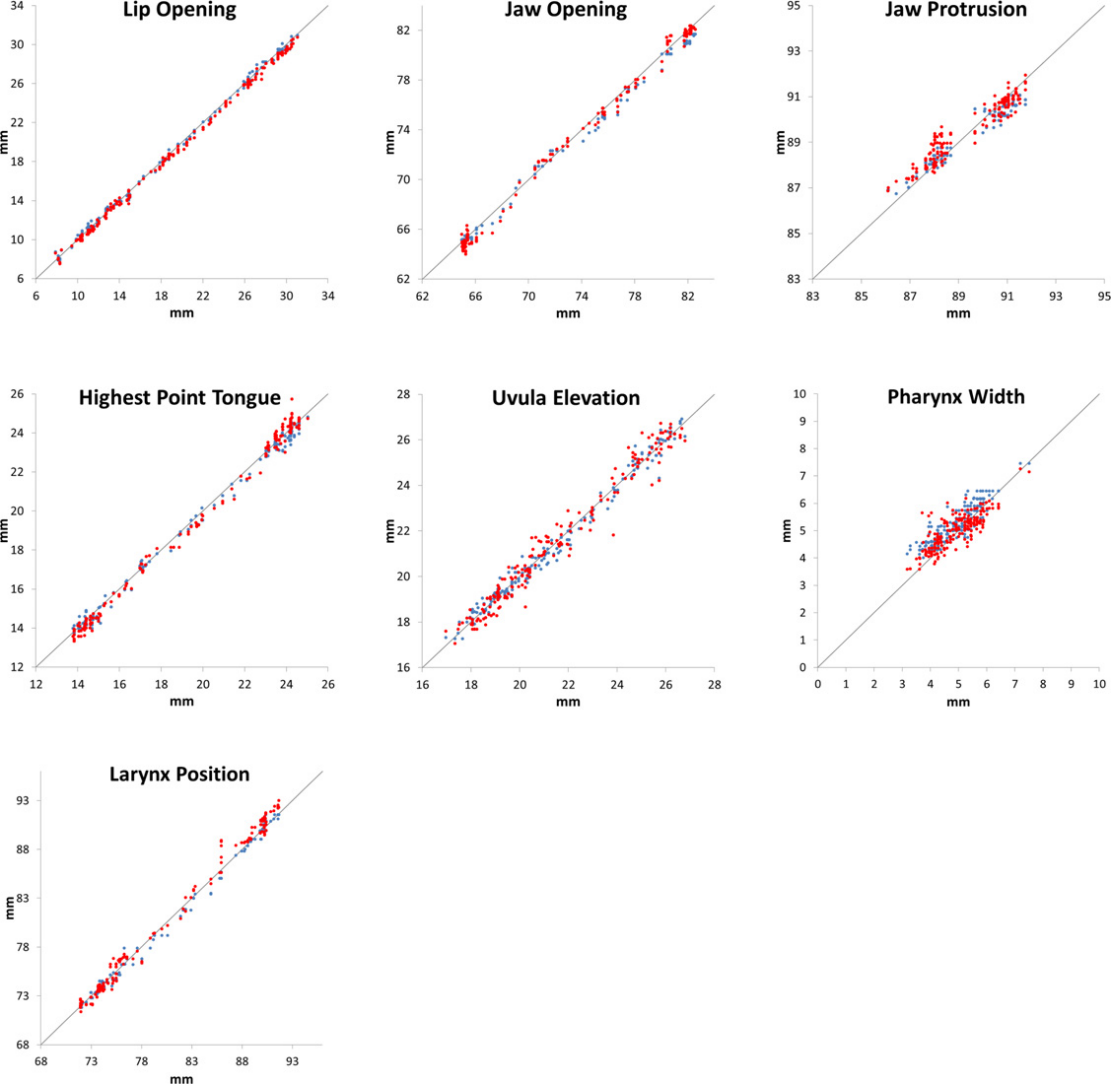
Scatter plots for all articulatory measures for subject 8, mf task. The original first rating is shown on the x-axis and the repeated measurements for the same rater (blue) and a different second rater (red), respectively, on the y-axis. The grey line refers to equivalence.

In order to estimate the error, repeated measurements were performed for the sequence of subject 8 in loudness condition mf. The subject was chosen randomly. Here, in order to estimate intra-rater agreement the same investigator evaluated the MRI data twice, with some time between the measurements. Furthermore, a second investigator measured the same articulatory parameters of the same sequence independent of the first investigator, in order to determine the inter-rater agreement. The results showed great consistency for all measured articulatory parameters both for the intra-rater and inter-rater-reliability (Fig 3). Table 2 provides the average absolute deviation from the mean (MAD) which is given in mm and in percent for both repeated measurements.

**Table 2:** Mean average deviation (MAD) in mm and % for the intra-rater and inter-rater difference. LO (Lip Opening), JO (Jaw Opening), JP (Jaw Protrusion) HPT (Highest Point Tongue), UE (Uvula Elevation), LP (vertical Larynx Position). The intra and inter-rater comparisons refer to the single subject 8, loudness mf.

	repeated measurements rater 1		rater 1 vs rater 2	
parameter	MAD [mm]	rel MAD [%]	MAD [mm]	rel MAD [%]
LO	.12	.72	.20	1.19
JO	.21	.28	.21	.29
JP	.19	.21	.20	.23
HPT	.12	.65	.15	.78
UE	.13	.60	.18	.85
PW	.19	3.83	.17	3.56
LP	.18	.23	.27	.34

### Statistical analysis

Statistical analyses were performed using SPSS 22 (SPSS Inc., Armonk, NY, USA). Independent analyses of variance (ANOVA) were used to investigate the differences between the three loudness conditions with respect to the measured articulatory parameters. Post-hoc tests (LSD) were performed on significant ANOVA results. The level of significance was set to p = 0.05. Correlations were reported as Pearson’s r coefficients. For the relation of pitch or ƒ_o_ and articulatory data, the relative scale degree was used. The scale degree has the advantage that it is insensitive to the fact that the scale used during the task was different between the voice classifications. The correlation was tested using the Kendall-Tau-b test.

## III. Results

**Fig 4:**
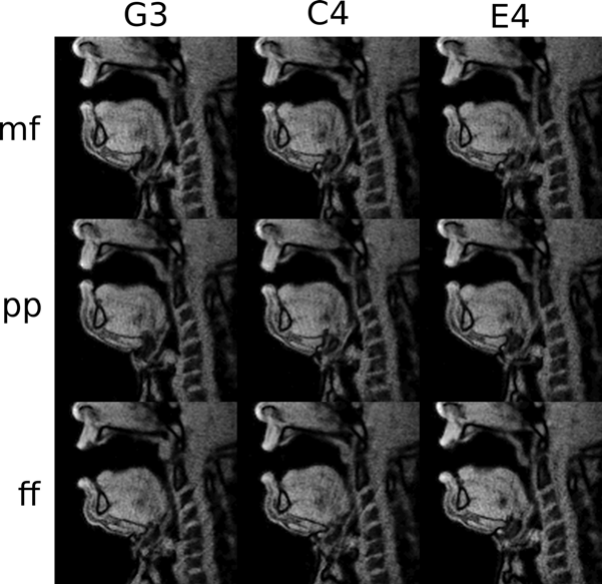
Representative mid-sagittal vocal tract profiles from a baritone (subject 10) for the different loudness tasks (mf = mezzoforte, upper row, pp = pianissimo middle row and ff = fortissimo, lowest row) for the pitches G3 (196Hz, left), C4 (262Hz, middle) and E4 (330Hz, right), respectively.

There were articulatory differences concerning the measurements associated with both a change in pitch and a difference in loudness. Fig 4 shows an example for the pitches G3 (196Hz), C4 (262Hz) and E4 (330Hz) for all three loudness conditions (mf, pp, ff, respectively in a baritone singer (subject 10). The entire MRI video sequence is provided in the supplementary video material (S1 Video).

**Fig 5:**
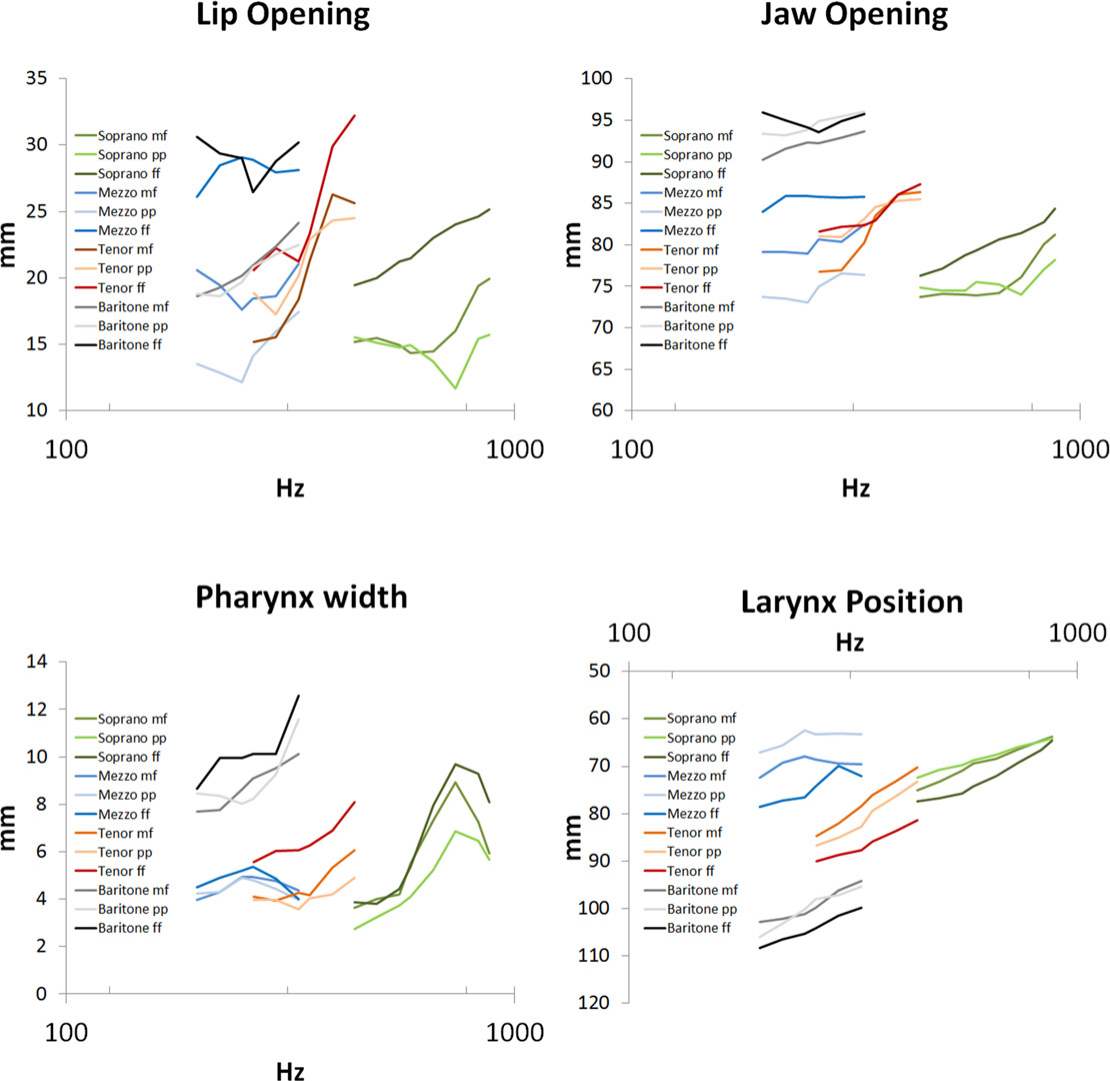
Mean values for the lip opening, jaw opening, pharynx width and larynx position, respectively, with respect to fundamental frequency (Hz). The green color refers to the sopranos, blue to mezzosopranos, red to tenors and grey to baritones. The differences in color intensity reflect the loudness condition: The darker the color, the louder the voice.

With rising pitch, lip opening and pharynx width were increased. Fig 5 presents these articulatory data with respect to ƒ_o_. Furthermore, with rising pitch there was an elevation of the vertical laryngeal position (Fig 5). As shown in Table 3, there were differences between the various voice classifications: While sopranos and tenors showed many correlations of articulatory data with the scale degree, there were almost no such correlations for the mezzosoprano voices.

**Table 3:** Kendall-Tau-b test for the description of the correlation between the scale degree and the articulatory data. The table shows data for the different voice classifications, soprano, mezzosoprano, tenor and baritone, separately.

		Scale degree				
		Soprano	Mezzo	Tenor	Bariton	All
**Lip Opening**	Kendall-Tau-b	.117	.069	.501^**^	.122	.120*
** **	Sig.	.167	.490	.000	.222	.011
**Highest Point Tongue**	Kendall-Tau-b	.070	-.045	.096	.043	.006
** **	Sig.	.410	.655	.336	.666	.897
**Uvula Elevation**	Kendall-Tau-b	.022	-.286^**^	-.025	.034	-.094*
** **	Sig.	.791	.004	.803	.733	.046
**Jaw Opening**	Kendall-Tau-b	.240^**^	.115	.248^*^	.108	.064
** **	Sig.	.005	.253	.013	.279	.172
**Jaw Protrusion**	Kendall-Tau-b	.234^**^	.105	.325^**^	.104	.003
** **	Sig.	.006	.292	.001	.299	.948
**Pharynx Width**	Kendall-Tau-b	.393^**^	-.008	.209^*^	.368^**^	.195**
** **	Sig.	.000	.934	.037	.000	.000
**Larynx Position**	Kendall-Tau-b	-.478^**^	-.107	-.469^**^	-.304^**^	-.233**
** **	Sig.	.000	.285	.000	.002	.000
**SPL**	Kendall-Tau-b	.511^**^	.067	.271^**^	.134	.294**
	Sig.	.000	.500	.007	.180	.000

*p<0.05

**p<0.01

**Fig 6:**
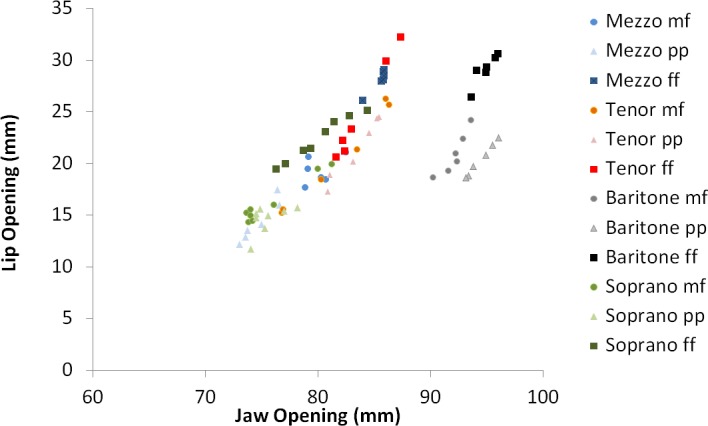
Jaw opening versus lip opening for the mean values across each fundamental frequency. The green color refers to the sopranos, blue to mezzo-sopranos, red to tenors and grey to baritones. The symbols reflect the loudness condition: circles = mf, triangles = pp, squares = ff.

The lip opening and jaw opening are not independent articulators: in general, if the jaw shows a greater opening also the lips revealed the same tendency (Pearson correlation: r = .663, p < .001, see Table 4). However, these articulators show also some degrees of independency. Fig 6 shows the jaw opening versus the lip opening. If, for a given jaw opening, the corresponding lip openings are compared between the different loudness conditions, it was found that those lip openings were greater in the loud phonation task.

**Table 4:** Pearson-correlation which indicates correlation between different articulatory variables and with SPL as well as statistical significances for all articulatory data.

		Lip Opening	Highest Point Tongue	Uvula Elevation	Jaw Opening	Jaw Protrusion	Pharynx Width	Larynx Position	SPL
**Lip Opening**	Pearson-correlation	1	.281**	.182**	.633**	.359**	.350**	.256**	.315**
	Sig.		.000	.005	.000	.000	.000	.000	.000
**Highest Point Tongue**	Pearson-correlation	.281**	1	.803**	.673**	.408**	.291**	.650**	.081
	Sig.	.000		.000	.000	.000	.000	.000	.219
**Uvula Elevation**	Pearson-correlation	.182**	.803**	1	.511**	.508**	.251**	.678**	-.076
	Sig.	.005	.000		.000	.000	.000	.000	.247
**Jaw Opening**	Pearson-correlation	.633**	.673**	.511**	1	.615**	.514**	.573**	.210**
	Sig.	.000	.000	.000		.000	.000	.000	.001
**Jaw Protrusion**	Pearson-correlation	.359**	.408**	.508**	.615**	1	.584**	.656**	.061
	Sig.	.000	.000	.000	.000		.000	.000	.356
**Pharynx Width**	Pearson-correlation	.350**	.291**	.251**	.514**	.584**	1	.405**	.387**
	Sig.	.000	.000	.000	.000	.000		.000	.000
**Larynx Position**	Pearson-correlation	.256**	.650**	.678**	.573**	.656**	.405**	1	.035
	Sig.	.000	.000	.000	.000	.000	.000		.597
**SPL**	Pearson-correlation	.315**	.081	-.076	.210**	.061	.387**	.035	1
	Sig.	.000	.219	.247	.001	.356	.000	.597	

*p<0.05

**p<0.01

**Fig 7:**
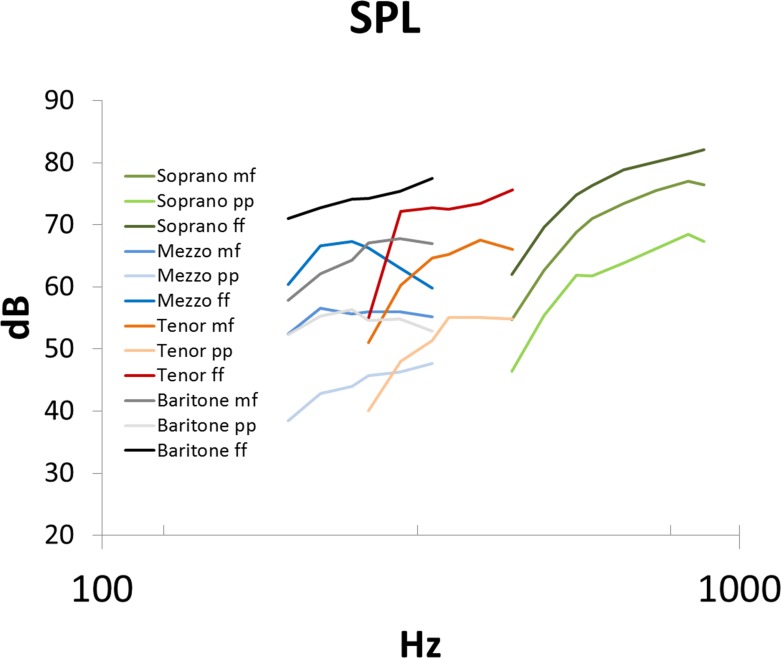
Mean values for sound pressure level (dB) with respect to fundamental frequency (Hz). The green color refers to the sopranos, blue to mezzo-sopranos, red to tenors and grey to baritones. The differences in color intensity reflect the loudness condition: the darker the color, the louder the voice.

With regard to different loudness conditions, the evaluation of the SPL showed that the perceptual loudness was associated with statistically significant differences of SPL, i.e. that pp showed the lowest, mf a medium, and ff showed the greatest SPL (mean values for pp = 53.3dB, mf = 63.6dB, ff = 71.3 dB, p<0.001) (Fig 7). The SPL was increased with pitch (Table 3).

The mean values of the articulatory data showed statistically different courses with regard to loudness for the lip opening (p<0,001), the jaw opening (p<0,01), the pharynx width (p<0,01) and the vertical laryngeal position (p<0,05). Here, the lip opening, the jaw opening and the pharynx width increased with loudness (Fig 5). Furthermore, the vertical laryngeal position was found to be lower for the louder phonation tasks (Fig 5). However, the changes in the jaw protrusion, uvula elevation, and highest point of the tongue failed to reach a statistical significance. In fact, concerning the uvula it was found that three of the subjects (subjects 5, 8 and 10) were changing the position of the uvula in such a way that the subjects opened their nasal cavities to the pharynx in the course of the tasks.

Since there were articulatory changes for both, the pitch and the SPL (Fig 5), it could be of interest how much these variables are correlated with the articulatory data. Table 3 shows the relation of the articulatory data with the scale degree and Table 4 with SPL. It was shown, that the correlation of the lip opening and the pharynx width with SPL was greater in comparison to the correlation with the scale degree.

## IV. Discussion

This study analyzes the effect of different loudness conditions on articulatory data concerning professional singers`singing voices. In general, articulatory changes were found for both increasing loudness and rising pitch.

Articulation is considered as an important factor which is changing with pitch [10–12,19,49]. The presented data are in agreement with previous MRI based studies which showed an increased lip opening, pharynx width and elevated vertical larynx position for rising pitches and ƒ_o_ [8–10]. Since a higher pitch and ƒ_o_ are frequently associated with a greater SPL and loudness [27], it could be expected that some of the articulatory data would also show a dependency on the SPL and loudness. However, the presented data also showed differences for the articulatory data when the same pitch in different loudness conditions was analyzed. Here, an increase of the SPL was associated with an opening of the lip and the jaw. Especially, the lip opening and pharynx width revealed a stronger correlation with the SPL than with the scale degree. Furthermore–although the lip and the jaw opening were in general strongly correlated–the lip opening corresponding to a given jaw opening was greater for the loudest task. It seems that the lip opening is sometimes additionally modified for the intended loudness condition. The greater lip and jaw opening cause a “trumpet shaped” vocal tract configuration. Furthermore, the increased jaw opening should raise the first resonance [27]. However, the effect of the lip opening on the first vocal tract resonance might be counteracted by the increased pharynx width. A greater pharynx width is likely to lower the first while increasing the second vocal tract resonance frequency [27,50]. The increased pharynx width could be related to the tongue position; however, the presented data failed to show a uniform tendency concerning the tongue height. Therefore, it could be expected that individual morphologies and singing techniques might also contribute to the articulation in this respect: most sopranos have smaller vocal tract relations than tenors or baritones [51] which might have influenced the data. This was the reason why the correlations of the articulatory data were compared to the relative scale degree instead of the absolute value of ƒ_o_. In order to compensate for anatomical differences, it could be worthwhile to analyze the articulatory differences in relation to an individual baseline in future investigations, such as the vocal tract length and width for the resting expiratory level. Lastly, while a vertical rise of the larynx was correlated with a rising pitch, the larynx was found at a lower vertical position in the louder dynamical conditions. This finding is in agreement with Luchsinger and Arnold [40] and Shipp [41] but in contrast to Neuschäfer-Rube et al. [46]. An elongation of the vocal tract will lower the vocal tract resonance frequencies. This phenomenon is often considered as *covered voice*, *voix sombrée* or *gedecktes Singen*. A lowering of the larynx will decrease the relation of the epilaryngeal tube to the pharynx cross section and will therefore also contribute to a clustering of resonances 3–5 [36]. The contrast of the presented data to Neuschäfer-Rube et al. [46] might be related to the fact that not all singers use formant/resonance clustering or that they accept timbre changes associated with an elongation of the vocal tract.

The control of the SPL and loudness is one of the most important parameters of voice production [27,32]. Since an increase of the subglottic pressure as a main factor for the increase of the SPL [27] is associated with an increase of the vocal fold impact stress and connected risk of vocal overuse [52], other strategies such as modification of the phonation type and vocal tract resonances might be of importance in order to increase the SPL and/or loudness. In this respect, the presented data show that the analyzed professional singers show differences in the vocal tract shape with regard to loudness, which might be due to resonatory strategies. In fact, much effort is given during singing voice education in order to learn such resonatory strategies. Therefore, the presented data might reflect such a training effect which might help the singers to produce a louder voice on stage without highly increasing the risk of overuse. In order to verify such a training effect, it seems relevant to analyze also untrained singing voices. However, up to now the MRI recording circumstances (supine position, singing in a tube, noisy surrounding etc.) still prevent a valid data acquisition and analysis for such untrained subjects. Furthermore, resonatory strategies might also be of importance for speech. In contrast to the singing voice, vowel production during speech is much faster and changing quickly due to prosody. Also, consonants are included in speech. As a consequence, it seems of interest for future investigations to analyze if the observed articulatory differences for the singing voice could also be detected in speech.

The presented data showed some differences concerning the vocal classifications. Compared to mezzos, particularly sopranos revealed stronger articulatory changes with rising pitch. In this respect, it should be noted here that these data are related to the vowel /a/ because it is considered as the vowel with the highest first formant [27]. Since articulatory changes are expected when ƒ_o_ reaches the region of the first formant [9,10,12,13] the vowel /a/ should be unproblematic for ƒ_o_s up to 700 Hz. As a consequence, in the presented experiment, the observed articulatory differences should be independent of the vowel condition for most voice classifications. It seems likely that other effects could be observed by using vowel conditions exhibiting lower first formant frequencies, such as /i/ or /u/. In the presented experiment only the sopranos were performing a task which reaches a ƒ_o_ greater than 700Hz. As a consequence, articulatory changes observed for these high pitches might also be related to the fact that singers tend to avoid the situation that ƒ_o_ crosses the first vocal tract resonance [28].

In previous studies it was found that register shifts from modal to falsetto were associated with only minor vocal tract shape changes, while the maintenance of the stage voice above the passaggio showed strong articulatory movements when reaching high pitches [8,10,15]. Since the falsetto is often associated with a lower SPL in comparison to the stage voice, part of the articulatory differences with respect to registers might also be related to loudness.

There are other limitations to the presented study. The subjects were asked to perform the task in different musically defined loudness conditions. Loudness is a subjective term. Although the examiners agreed that the different loudness conditions were fulfilled by the subjects, such perceptual evaluation should be considered problematic due to the noisy surroundings. Therefore, in the present experiment an attempt was made to measure the SPL after noise cancellation. Here, for every subject it was found that for every pitch pp was associated with the lowest, mf with medium, and ff with the greatest SPL, respectively. Since noise reduction was performed by means of dedicated software the noise cancellation process may also have changed the voice signal. Furthermore, since there are other approaches for noise cancellation [53,54] it cannot be excluded that other systems would show differences concerning the absolute values of the SPL. However, the same filter conditions were applied to all three of the tasks (pp, mf, and ff, respectively) and the error should only be of a systematic nature. Furthermore, as can be observed from the audio-files, the noise cancelled sound was considered perceptually quite satisfactory. There is another limitation concerning the presented evaluation of the SPL: the recordings were performed in a noisy environment which was only in part diminished by using ear-protective headphones. Although singers might be rather indifferent to noise by their professional training, it cannot be totally excluded that these classically trained subjects raised their sound pressure level with increasing noise, a phenomenon which is often denoted as the Lombard effect [55,56].

The recordings were performed in a supine position. Although Traser et al. [57] found nearly no articulatory differences concerning vocal tract shapes while singing in upright versus supine position in a study with professional singers, the authors found a higher vertical larynx position for the supine position. Therefore, it could be expected that the vertical laryngeal position could be even lower for singing in the (more natural) upright position.

The task for the mezzo sopranos was chosen at a rather low pitch in order to achieve comparability to male altos analyzed in a previous study [17]. Here, articulatory changes with respect to pitch showed comparable tendencies to male altos when these subjects maintained their modal register, avoiding changes to their stage falsetto. Only the pharynx width which was much increased for the male altos during rising pitch showed no such tendency for the mezzos. Due to the relatively low pitch it cannot be excluded that mezzo sopranos would show different articulatory behavior at higher pitches.

The presented data refer to classically trained singers. However, there are other professional singers singing in non-classical styles: it was shown in a pilot study that vocal tract shape might differ between a more classical way of singing in comparison to belting in Musical Theatre singing [19]. Frequently, belting is associated with a greater SPL [58–60]. How much articulatory data are influenced by loudness and SPL in this special style of phonation should be clarified by further research.

## V. Conclusions

Loudness has an effect on articulation in professional singers`singing voices for the vowel /a/, which should be considered when articulatory vocal tract data are interpreted. If the observed effects are vowel-dependent and if loudness also contributes to articulatory data in speech is a matter of future research.

## Supporting Information

S1 Audio Audio before the Adobe Audition filtering process. (WAV)

S2 Audio Audio after the Adobe Audition filtering process. (WAV)

S1 Table MRI raw data excel file. (XLSX)

S1 Video Subject 10 singing the task in mf, pp, and ff. (MP4)
